# Dementia prediction in the general population using clinically accessible variables: a proof-of-concept study using machine learning. The AGES-Reykjavik study

**DOI:** 10.1186/s12911-023-02244-x

**Published:** 2023-08-28

**Authors:** Emma L. Twait, Constanza L. Andaur Navarro, Vilmunur Gudnason, Yi-Han Hu, Lenore J. Launer, Mirjam I. Geerlings

**Affiliations:** 1 Department of Epidemiology, Julius Center for Health Sciences and Primary Care, University Medical Center Utrecht and Utrecht University, Utrecht, the Netherlands; 2 Department of General Practice, Amsterdam UMC, location Vrije Universiteit Amsterdam, De Boelelaan 1117, Amsterdam, the Netherlands; 3 Amsterdam Public Health, Aging & Later life and Personalized Medicine, Amsterdam, the Netherlands; 4 Amsterdam Neuroscience, Neurodegeneration and Mood, Anxiety, Psychosis, Stress, and Sleep, Amsterdam, the Netherlands; 5 Faculty of Medicine, University of Iceland, Reykjavik, Iceland; 6 The Icelandic Heart Association, Kopavogur, Iceland; 7 Laboratory of Epidemiology and Population Sciences, National Institute on Aging, Baltimore, MD USA; 8 Department of General Practice, Amsterdam UMC, location University of Amsterdam, Meibergdreef 9, Amsterdam, the Netherlands

**Keywords:** Dementia, Machine learning, Prediction model

## Abstract

**Background:**

Early identification of dementia is crucial for prompt intervention for high-risk individuals in the general population. External validation studies on prognostic models for dementia have highlighted the need for updated models. The use of machine learning in dementia prediction is in its infancy and may improve predictive performance. The current study aimed to explore the difference in performance of machine learning algorithms compared to traditional statistical techniques, such as logistic and Cox regression, for prediction of all-cause dementia. Our secondary aim was to assess the feasibility of only using clinically accessible predictors rather than MRI predictors.

**Methods:**

Data are from 4,793 participants in the population-based AGES-Reykjavik Study without dementia or mild cognitive impairment at baseline (mean age: 76 years, % female: 59%). Cognitive, biometric, and MRI assessments (total: 59 variables) were collected at baseline, with follow-up of incident dementia diagnoses for a maximum of 12 years. Machine learning algorithms included elastic net regression, random forest, support vector machine, and elastic net Cox regression. Traditional statistical methods for comparison were logistic and Cox regression. Model 1 was fit using all variables and model 2 was after feature selection using the Boruta package. A third model explored performance when leaving out neuroimaging markers (clinically accessible model). Ten-fold cross-validation, repeated ten times, was implemented during training. Upsampling was used to account for imbalanced data. Tuning parameters were optimized for recalibration automatically using the caret package in R.

**Results:**

19% of participants developed all-cause dementia. Machine learning algorithms were comparable in performance to logistic regression in all three models. However, a slight added performance was observed in the elastic net Cox regression in the third model (*c* = 0.78, 95% CI: 0.78–0.78) compared to the traditional Cox regression (*c* = 0.75, 95% CI: 0.74–0.77).

**Conclusions:**

Supervised machine learning only showed added benefit when using survival techniques. Removing MRI markers did not significantly worsen our model’s performance. Further, we presented the use of a nomogram using machine learning methods, showing transportability for the use of machine learning models in clinical practice. External validation is needed to assess the use of this model in other populations. Identifying high-risk individuals will amplify prevention efforts and selection for clinical trials.

**Supplementary Information:**

The online version contains supplementary material available at 10.1186/s12911-023-02244-x.

## Introduction

Dementia is characterized by debilitating cognitive impairment that increases the risk of mortality [1], while quality of life decreases for both the patient and his or her caregivers. Currently, 50 million people in the world have dementia, which is expected to triple by 2050 [2]. While much research has been done on the risk factors for dementia, no effective treatment is available [3]. Further, by the time of diagnosis, the brain has already substantially declined in function [4]. Thus, early classification is crucial for prompt intervention and better outcomes for high-risk individuals. Many prognostic models for incident dementia have been developed using ‘traditional’ statistical techniques, such as logistic or Cox regression [5–8]. However, external validation of these models showed poor calibration and performance [9, 10], highlighting the need for updated models for prognostication of dementia. The recent increased application of machine learning for disease prediction offers the possibility to improve dementia prognostic models. Machine learning can aid in unraveling complex relationships between predictors, taking into account nonlinear relationships and interactions, while additionally using that information to increase a model’s predictive performance [11].

Research thus far using machine learning for dementia prediction is in its infancy and current models primarily focus on magnetic resonance imaging (MRI) for prediction (please see these recent reviews for an overview [12–14]). Some studies have explored demographic factors [15, 16] and plasma proteomic data [17–19], but no studies have yet also explored some commonly assessed biomarkers (e.g., glucose, cholesterol, blood pressure) along with demographic and lifestyle information in dementia prediction using machine learning classifiers [12]. A recent review also highlighted the need for the development of new prognostic models for dementia that focus on clinical variables over imaging variables [12]. An emphasis on predictors that are more clinically accessible than MRI is crucial for the potential future use of prognostic models for dementia in clinical practice. Focusing on accessible predictors will allow for wider generalizability of the assessment of high-risk individuals for dementia into the general population. It follows the order and flow of the diagnostic process, by focusing first on cheaper, less invasive, and potentially more accessible predictors in a general practice setting, the starting point for a patient, as opposed to in a memory clinic.

Previous studies using machine learning methods have mostly used the Alzheimer’s Disease Neuroimaging Initiative (ADNI) cohort for algorithm testing [12], with relatively limited sample sizes (i.e., less than 1,000 participants). Discrimination has focused on differentiating mild cognitive impairment [15] from Alzheimer’s disease [12], the leading cause of dementia. Further, most studies that implemented machine learning methods did not take class imbalance into account [12], which focuses on negative predictive value over positive predictive value and introduces possible bias. As previous studies have also focused on cohorts that have more cases than controls, the possible generalizability of the prognostic model decreases [14]. Therefore, there is a current gap in developing a dementia risk model using machine learning for the general population, using a large sample size.

Our research questions were the following: (1) What is the added performance of machine learning algorithms (i.e., elastic net regression, random forest, support vector machine) for dementia prognosis compared to traditional statistical techniques (e.g., logistic and Cox regression) in a large, population-based cohort from Reykjavik, Iceland of almost 5,000 individuals without dementia or mild cognitive impairment (average age: 76 years, 69% female, 29% with college/university level education)? (2) What is the difference in performance when focusing only on clinically accessible predictors? (3) What is the difference in performance when assessing women and men separately?

## Methods

This study was reported following the Transparent Reporting of a Multivariable Prediction Model for Individual Prognosis or Diagnosis (TRIPOD) Statement [20].

### Study sample

Data originated from the Age, Gene/Environment Susceptibility (AGES)-Reykjavik Study, a community-based cohort study of individuals 65 years or older living in the Reykjavik area. More details are provided elsewhere [21]. In brief, participants from the AGES-Reykjavik Study stem from the Reykjavik study, initiated in 1967 by the Icelandic Heart Association. Between 2002 and 2006, 5,764 individuals randomly selected from survivors of the Reykjavik Study were included. Baseline cognitive, biometric, and MRI assessments were done at the Reykjavik research center. Individuals with dementia or mild cognitive impairment at baseline were excluded from the current analysis, leaving 4,793 individuals in the analytical sample. Cognitive, biometric, and MRI assessments were done at baseline between 2002 and 2006, with follow-up of incident dementia diagnoses for a maximum of 12 years. Written informed consent was obtained from all participants. The Icelandic National Bioethics Committee (VSN: 00–0063), the Icelandic Data Protection Authority, and the Institutional Review Board for the National Institute on Aging, NIH approved this study.

### Dementia assessment

Details regarding the procedure for dementia ascertainment can be found elsewhere [22–24]. In brief, a three-step procedure based on international guidelines [21] was used. First, all participants underwent neuropsychological testing of cognition using the Mini-Mental State Examination (MMSE) and the Digit Symbol Substitution Test [23], with the next step in those who screened positive undergoing further neuropsychological examination. In the third step, in those who screened positive on the neuropsychological examinations, further proxy and diagnostic assessments were performed regarding the Activities of Daily Living (ADL), as well as social and cognitive functioning. Then, a multidisciplinary panel including a neurologist, geriatrician, neuroradiologist, and neuropsychologist performed a consensus diagnosis that included exam measures and brain MRI [24]. Additional dementia cases were also obtained through medical and nursing home records as well as in death certificates. Dementia cases obtained through nursing homes were collected following a standardized protocol in Icelandic nursing homes [25]. The current study focused on all-cause dementia only.

### Demographics

Age (continuous), sex (dichotomous), education (categorical; categorized as primary school, secondary school, college, or university), and current marital status (married/living together, widowed, divorced, single) were collected by questionnaire at baseline.

### Clinical variables

A wide range of clinical variables were used, including metabolic, lipid, and inflammatory levels, as well as medical diagnoses (more information in Supplementary Info 1).

### Medication use

Medication use was treated as dichotomous (yes/no) for benzodiazepines, beta-adrenergic blockers, glucocorticoids, psycholeptics, or anti-depressants.

### Lifestyle variables

We included the following continuous variables: alcohol consumption, mental leisure activity (days per month), social leisure activity (days per month), number of close friends, and number of living close relatives. The categorical variables we included are as follows: smoking status (current, former, never), physical activity within the last 12 months (never, rarely, occasionally, moderate, high), difficulty in walking 2 km (very easy, somewhat easy, not that easy), difficulty in walking 500 m (very easy, somewhat easy, not that easy), and how often fish is consumed as the main meal (never, less than once a week, 1–2 times a week, 3–4 times a week, 5–6 times a week, daily, more than once a day).

### Cognitive assessment

The raw total score of the test of global cognitive function, the MMSE, was the only variable used to assess cognition.

### Neuroimaging variables

MR images were collected using 1.5T brain MRI (Signa TwinSpeed; General Electric Medical Systems). For more information on the MRI protocol, refer to [26–28]. Log-transformed white matter lesion volume and hippocampal volume, as well as the ratio of gray matter/intracranial volume (to account for correlation), and the number of cerebral microbleeds were entered as continuous predictors. The presence of infarcts (yes/no) was entered as a dichotomous variable.

### Statistical analyses

All analyses were performed in R (v 4.0.3). Before beginning the analyses, data were split into a two-thirds (proportion: 0.66) training set and a one-third test set, ensuring for balanced incident dementia cases in the train/test sets by using the split_df() function in R.

### Sample size calculations

We performed a post-hoc sample size calculation using *pmsampsize* package in R to calculate the number of events/cases required using logistic regression as best-case-scenario [29]. If all predictors are included, the required sample size is at least 1,691, which is less than the current sample of 4,793.

### Missing data

Half of the individuals (55%) had at least one missing value on predictors (max: 27% missing on ability to walk 2 km or 500 m). There were no missing values on the outcome (i.e., dementia). Missing data were handled with multiple imputation using the *mice* package in R separately in the training and test sets using ten imputed datasets. The predictor matrix for the training set was used for imputation in the test set. All predictors as well as the outcome were used in the imputation process. A random imputed dataset from a total of ten was selected for further analyses for both the training and test sets as pooling methods for machine learning prognostic models have yet to be validated. See Supplementary Table 1 for an overview of predictors and outcome in both training and test sets.

### Model building

The *caret* package in R [30] was used for all prediction models, i.e. elastic net regression, random forest, support vector machine, and logistic regression. To take time-to-event and censoring into account, we also performed a regular Cox regression using the *glmnet* package [31] and elastic net Cox regression using the *hdnom* package [32] in R. For the support vector machine classifier, a radial kernel was used to allow for nonlinear separations of the data. Hyperparameter tuning was performed automatically by caret. Pseudocode can be found in Supplementary Code 1. The models were first fitted with all features (model 1). Then, models were fit after feature selection using the *Boruta* package in R [33] for more parsimonious models (model 2). In short, Boruta uses a random forest classifier and applies mean decrease accuracy to evaluate each feature’s importance based on 99 iterations. Tentative features were not included. Lastly, to evaluate a clinically accessible model (i.e., one that does not include MRI features), models were fit only with features selected from Boruta that were not MRI (model 3). Tuning parameters were optimized for recalibration and varied across all three models (Supplementary Table 2).

### Internal validation

Using cross-validation, more variability is introduced into the training of each classifier. Ten-fold cross-validation, repeated ten times, for a total of 100 times, was used in training each machine learning algorithm. The training data are divided into ten folds, with the given classifier trained on nine folds, using the tenth for testing. This is repeated until each of the ten folds is held back for testing. The performance metrics are then averaged across all repetitions. Further, upsampling was performed to handle imbalanced data and was implemented during cross-validation. This is done by resampling with replacement our class with incident dementia (i.e., the minority class) to be the same size as those who do not develop dementia (i.e., the majority class). If models failed to converge with upsampling, downsampling was used, which deletes samples from the majority class (i.e., those who do not develop dementia). Additionally, we tested different thresholds for classification other than 0.5, ranging from 0.10 to 0.90 by steps of 0.02.

### Performance metrics

The following performance measures were used to assess the models: area under the receiver operating characteristic (ROC) curve (AUC), sensitivity, specificity, positive predictive value, and negative predictive value. The model with the highest AUC was then used for the test set. For the survival models, the *c*-statistic was used. *C*-statistics and AUC values are comparable to assess performance. The *MLeval* package in R was used to calculate 95% confidence intervals. Bootstrapping using the *hdnom* package was done to calculate 95% confidence intervals in the elastic net Cox regression models. The *hdnom* package was used to create calibration plots for the elastic net Cox regression as well as to create a clinically relevant nomogram.

### Sensitivity analysis

To assess if the prognostic model has similar performance in men and women, the trained model in both sexes was tested on men and women separately.

## Results

During an average of 9 ± 3 years of follow-up, 892 (*n* = 750 from nursing homes) individuals developed dementia. Mean (SD) age at baseline for all participants was 76 [6] years and 59% were female. Demographic and clinical information for the full study sample on all predictor variables and the outcome are shown in Table 1.

**Table 1:** Characteristics of the predictors in the study sample (n = 4793)

	Mean (SD) or n (%)	% missing per variable
**Demographics**		
Age (years)* ^+^	76 (6)	0%
Sex (female)* ^+^	2822 (59%)	0%
Education (college + university)	1392 (29%)	6%
**Neuroimaging variables**		
Log-transformed white matter lesion volume (ml)*	13.5 (2.5)	18%
Hippocampal volume (ml)*	5.6 (0.7)	17%
Number of microbleeds*	0.3 (1.6)	17%
Presence of infarcts	1491 (31%)	16%
Gray matter volume (ml)*	676 (63)	18%
Intracranial volume (ml)*	1501 (148)	18%
**Clinical variables**		
Abdominal circumference (cm)	101 (12)	1%
Carotid intima-media thickness test (CIMT)	1 (0.1)	10%
High-density lipoprotein (mmol/L)	1.6 (0.5)	< 1%
Low-density lipoprotein (mmol/L)	3.5 (1.0)	< 1%
Triglycerides (mmol/L)	1.2 (0.7)	< 1%
Fasting glucose (mmol/L)	5.8 (1.2)	< 1%
B-hemoglobin A1c (g/dl)	0.5 (0.1)	8%
High-sensitive c-reactive protein (mg/L)	3.8 (6.7)	< 1%
Systolic blood pressure (mmHg)	142 (21)	1%
Diastolic blood pressure (mmHg)	74 (10)	1%
Hypertension	3855 (80%)	1%
Coronary artery disease	842 (18%)	0%
Diabetes mellitus	591 (12%)	0%
Metabolic syndrome	1499 (31%)	1%
Stroke/blood clot in the brain	297 (6%)	2%
History of cancer	753 (16%)	1%
Experienced a head trauma or lost consciousness	416 (9%)	5%
Subjective memory decline*^+^	1431 (30%)	3%
Often forget the names of a friend	1522 (32%)	5%
Often forget where items are*^+^	2083 (44%)	5%
Difficulty finding the right words	1517 (32%)	5%
Difficulty finding the way to familiar places*^+^	385 (8%)	5%
Inability in managing money*^+^	132 (3%)	4%
Inability in dressing oneself*^+^	29 (1%)	6%
Intermit claudication in legs	227 (5%)	5%
Insomnia	1438 (30%)	3%
Poor health status	276 (6%)	1%
ADL score, full dependence on all items*^+^	52 (1%)	6%
Morning salivary cortisol (nmol/L)	19.8 (13.3)	9%
Evening salivary cortisol (nmol/L)	3.8 (6.6)	9%
GDS-15 sum score*^+^	2.3 (2.1)	6%
All anxiety questions ‘yes’	40 (1%)	1%
Diagnosis of current GAD, social phobia, panic disorder, or agoraphobia	98 (2%)	5%
Current/past diagnosis of major depressive disorder	248 (5%)	5%
**Medication use**		
Benzodiazepines	396 (8%)	0%
Beta-adrenergic blockers	1660 (35%)	0%
Glucocorticoids	171 (4%)	0%
Psycholeptics	818 (17%)	0%
Anti-depressants	662 (14%)	0%
**Lifestyle variables**		
Current smoker, %	582 (12%)	4%
Alcohol consumption (g/week)	16 (33)	4%
Moderate/high physical activity	1509 (31%)	7%
Mental leisure activity (days per month)	7 (6)	6%
Social leisure activity (days per month)	4 (4)	6%
Single marital status, %	288 (6%)	6%
Number of close friends	4 (4)	6%
Not that easy to walk 2 km*^+^	960 (20%)	27%
Not that easy to walk 500 m*^+^	233 (5%)	27%
Number of living close relatives	7 (4)	6%
Never fish consumption, %	26 (1%)	6%
**Cognitive assessment**		
MMSE total score*^+^	27 (3)	1%
**Outcome**		
Incident dementia	892 (19%)	0%
Follow-up time (years)	9 (3)	0%

* marks variables entered in model 2. ^+^ marks variables entered in model 3. GAD = generalized anxiety disorder. GDS-15 = Geriatric Depression Scale-15. CVLT = California Verbal Learning Test

### Model performance

Logistic regression (AUC = 0.73, 95% CI: 0.71–0.75) had a similar AUC to the elastic net regression (AUC = 0.74, 95% CI: 0.72–0.76) and random forest classifiers (AUC = 0.74, 95% CI: 0.72–0.76) in model 1 (i.e., the full model), as well as in the model after feature selection and after removal of neuroimaging variables (Table 2). Support vector machine showed lower performance compared to all other machine learning classifiers and the logistic regression. Both logistic regression and the elastic net regression had the same performance in model 3 without neuroimaging variables (AUC = 0.71, 95% CI: 0.68–0.74) (Table 2).

**Table 2:** Summary of cross-validated prediction models on trained data (n = 3473)

Model	AUC	Sensitivity (%)	Specificity (%)	PPV (%)	NPV (%)
*Model 1*					
Logistic regression	0.73 [0.71–0.75]	64 [60–68]	70 [68–71]	32 [30–35]	89 [88–91]
Elastic net	0.74 [0.72–0.76]	68 [64–71]	69 [67–71]	33 [31–36]	90 [89–92]
Random forest	0.74 [0.72–0.76]	6 [4–8]	99 [99–99]	60 [47–71]	82 [81–83]
SVM	0.65 [0.62–0.68]	49 [45–53]	73 [71–74]	29 [27–32]	86 [85–88]
*Model 2*					
Logistic regression	0.74 [0.72–0.76]	67 [63–70]	70 [68–72]	34 [31–36]	90 [89–91]
Elastic net	0.74 [0.72–0.76]	67 [63–70]	69 [67–71]	33 [30–36]	90 [89–91]
Random forest	0.74 [0.72–0.76]	47 [43–51]	84 [82–85]	40 [36–44]	88 [86–89]
SVM	0.73 [0.71–0.75]	72 [69–76]	63 [61–65]	31 [28–33]	91 [89–92]
*Model 3*					
Logistic regression	0.71 [0.68–0.74]	64 [60–68]	68 [66–70]	31 [29–34]	89 [88–91]
Elastic net	0.71 [0.68–0.74]	64 [60–67]	67 [65–69]	31 [28–33]	89 [88–90]
Random forest	0.71 [0.68–0.74]	55 [51–59]	75 [73–77]	34 [31–37]	88 [87–89]
SVM	0.70 [0.67–0.73]	69 [65–73]	61 [59–63]	29 [27–31]	90 [88–91]

AUC = area under the ROC curve. SVM = support vector machine

**Fig. 1:**
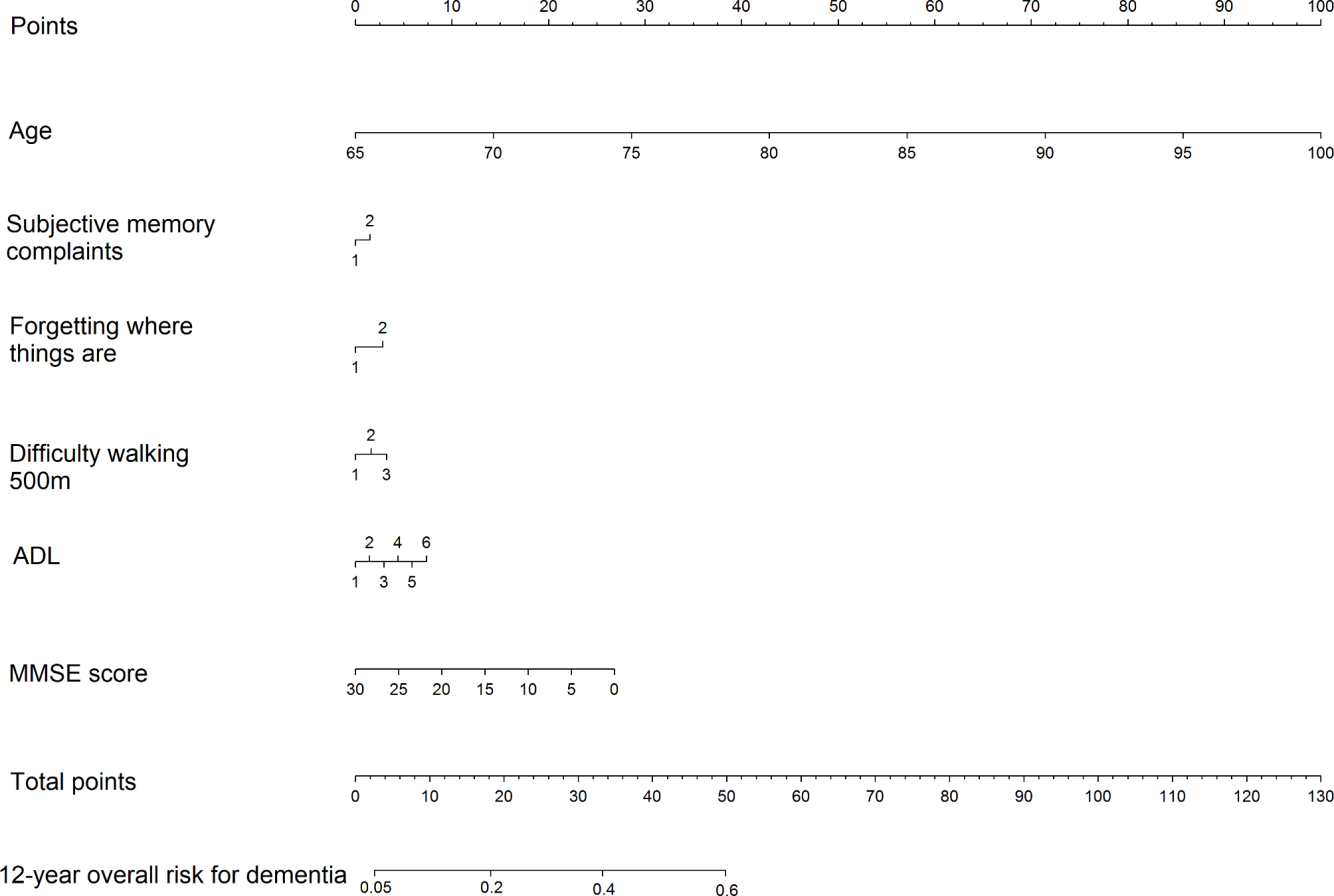
Predictive nomogram for 12-year overall risk for incident dementia in the elastic net Cox regression for model 3. To predict the patient’s risk for dementia, one can draw a vertical line to the top given each variable to get the number of points per that variable. The points from each variable are then summed and the total number of points represents a patient’s overall 12-year risk

When taking time-to-event into account with the elastic net Cox model, the *c*-statistic was high (*c* = 0.80, 95% CI: 0.79–0.80) in model 1 and higher than the traditional Cox model (*c* = 0.78, 95% CI: 0.77–0.79). The same *c-*statistics and confidence intervals were seen in model 2. Performance slightly lowered in model 3, but the elastic net Cox regression still showed higher *c*-statistics (*c* = 0.78, 95% CI: 0.78–0.78, model 3) compared to the traditional Cox model (*c* = 0.75, 95% CI: 0.74–0.77). The results of the elastic net Cox regression for model 3 are presented as a nomogram in Fig. 1 for 12-year overall risk. To predict the patient’s risk for dementia, one can draw a vertical line to the top given each variable to get the number of points per that variable. The points from each variable are then summed and the total number of points is used to give a patient’s overall 12-year risk.

When testing different thresholds, all classifiers demonstrated optimal sensitivity and specificity at 0.50.

Regarding resampling, up-sampling was used for all models except for all support vector machine models. Down-sampling was used instead for model convergence.

### Feature selection

For feature selection, Boruta ranked the following variables as most important: age, hippocampal volume, log-transformed white matter lesion volume, gray matter/intracranial volume ratio, MMSE score, difficulty finding the way to familiar places, difficulty in dressing oneself, subjective memory decline, the ADL score, forgetting where items are, number of microbleeds, the sum score of the Geriatric Depression Scale-15, how difficult it is to walk 500 m, sex, inability to manage money, and how difficult it is to walk 2 km (Supplementary Fig. 1). These variables were then used as the predictors in the parsimonious model (model 2), and then the MRI variables were removed for the clinically accessible model (model 3).

Variable importance slightly differed per algorithm in model 3. The least amount of variables used were in the elastic net regression (Supplementary Fig. 2). As there is no built-in variable importance for support vector machine, the AUC is shown instead on the x-axis.

### Internal validation

As the elastic net model performed the best regarding AUC, sensitivity, and specificity, it was chosen as the classifier to be used on the test data. The AUC was the same for both models 1 and 2 (AUC = 0.73; 95% CI: 0.70–0.76) and slightly decreased in model 3 when MRI variables were removed (AUC = 0.72; 95% CI: 0.69–0.75) (Table 3). Sensitivity was the same in all models (Sensitivity = 61%; 95% CI: 56–66%), and specificity was highest in model 2 (Specificity = 71%; 95% CI: 69–74%) (Table 3). For the elastic net Cox model, *c-*statistics were comparable for all three models (model 3: *c* = 0.77; 95% CI: 0.77–0.78).

**Table 3:** Summary of the elastic net models on test data (n = 1870), as well as stratified by sex

	AUC	Sensitivity (%)	Specificity (%)	PPV (%)	NPV (%)
**Model 1**	0.73 [0.70–0.76]	61 [55–66]	71 [69–73]	33 [29–37]	89 [87–91]
Women	0.74 [0.70–0.78]	60 [53–67]	70 [67–73]	35 [30–40]	87 [84–89]
Men	0.73 [0.67–0.79]	64 [55–73]	71 [67–75]	29 [23–35]	92 [89–94]
**Model 2**	0.73 [0.70–0.76]	61 [56–66]	71 [69–74]	33 [29–37]	89 [87–91]
Women	0.73 [0.69–0.77]	59 [52–66]	71 [67–74]	35 [30–40]	87 [84–89]
Men	0.73 [0.67–0.79]	63 [54–72]	72 [68–76]	29 [24–36]	92 [89–94]
**Model 3**	0.72 [0.69–0.75]	61 [56–66]	69 [66–71]	31 [28–35]	89 [86–90]
Women	0.71 [0.67–0.75]	59 [52–65]	69 [66–72]	33 [29–38]	86 [84–89]
Men	0.72 [0.66–0.78]	66 [57–75]	67 [63–71]	27 [22–33]	92 [89–94]

AUC = area under the ROC curve; PPV = positive predictive value; NPV = negative predictive value

### Calibration

**Fig. 2:**
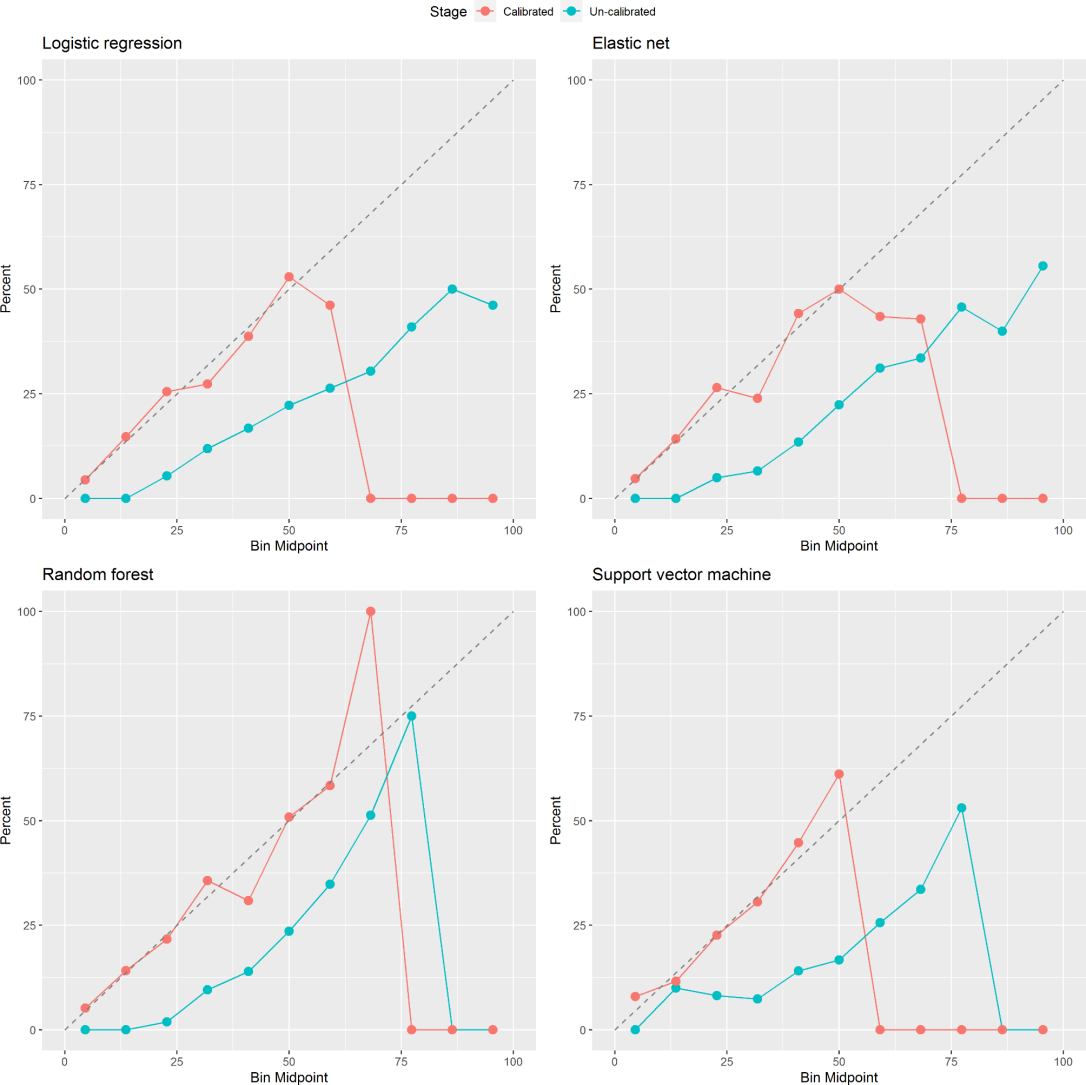
Calibration plots for logistic regression, elastic net regression, random forest, and support vector machine in model 3 (clinically accessible model) both before and after recalibration. Performance above the diagonal represents under-forecasting and performance below the diagonal represent over-forecasting. There were no individuals in the bins after 77

**Fig. 3:**
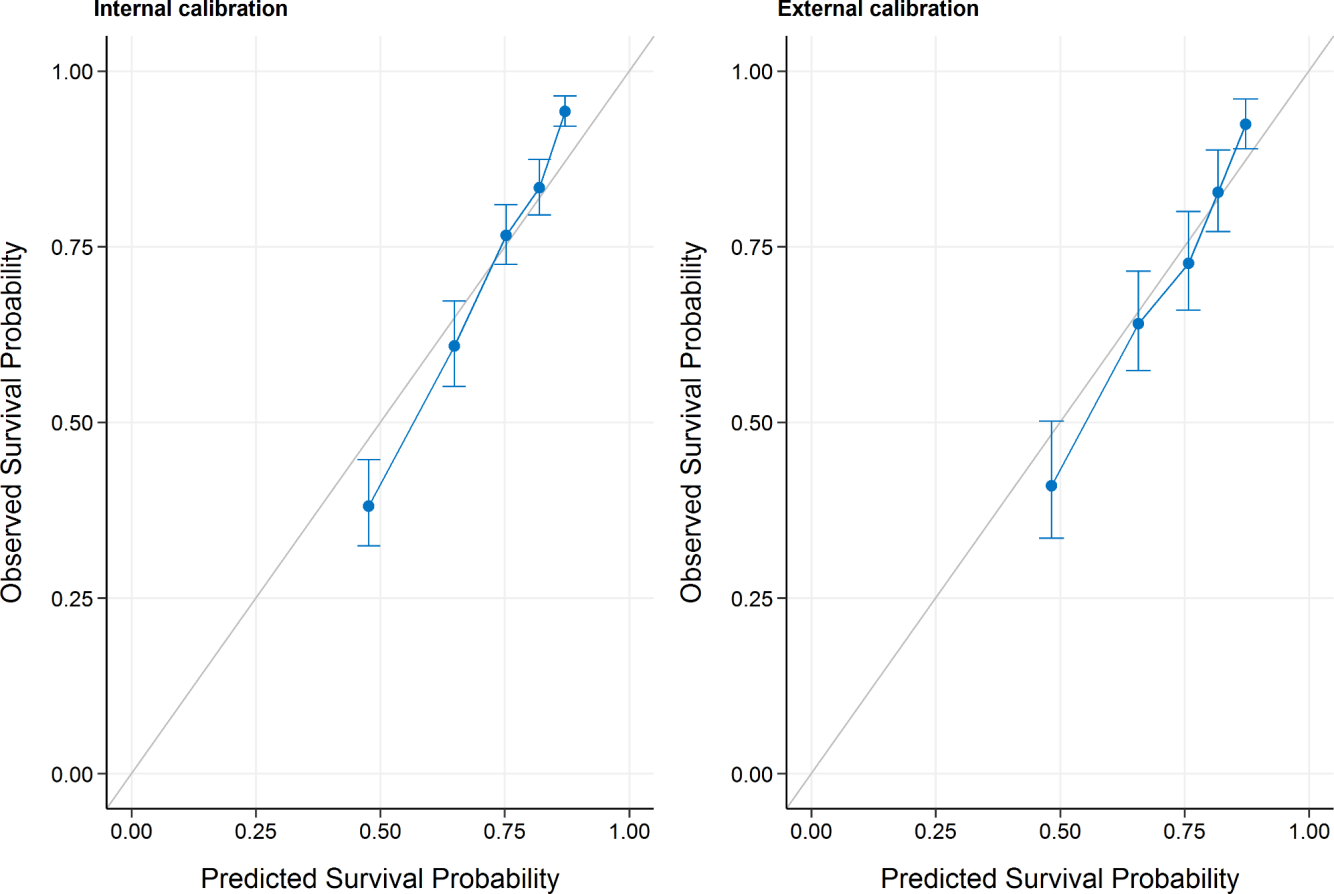
Calibration plots for the elastic net Cox regression in both the training set (internal calibration) and in the test set (‘external’ calibration). Performance above the diagonal represents under-forecasting and performance below the diagonal represents over-forecasting

Calibration was assessed for all models. All models showed overfitting, which was resolved after re-calibration (Fig. 2). Re-calibration was performed by training a logistic regression using the uncalibrated probabilities as a predictor. In the elastic net Cox regression, calibration was optimal in both our training (internal calibration) and testing sets (external calibration) (Fig. 3).

#### Sex stratification

Models were also tested on women only and men only to assess possible differences in predictive accuracy when stratified by sex. Across all models using elastic net regression, men and women had similar AUCs. Sensitivity was slightly higher in men, whereas specificity was slightly higher in women (Table 3). However, confidence intervals overlapped. In the elastic net Cox regression model, men (*c* = 0.86, 95% CI: 0.85–0.87, model 3) had higher *c-*statistics than women (*c* = 0.73, 95% CI: 0.72–0.74, model 3) in all three models.

## Discussion

The current study aimed to explore the difference in performance between machine learning algorithms and traditional statistical methods for a prognostic model for dementia. We further aimed to assess the feasibility of only using clinically accessible predictors compared to including structural brain MRI, as well as exploring model performance when stratifying by sex. Machine learning only showed benefit over traditional statistical methods when using survival methods. When removing imaging variables from the prediction model, AUC and *c-*statistic values slightly lowered but remained high. Models performed similarly in men and women in the elastic net regression; however, in the elastic net Cox regression, men had higher *c*-statistics compared to women.

The current study explored the difference in performance when using machine learning methods compared to traditional statistical techniques. Previous prediction models using machine learning yielded high performance accuracy when using only MRI variables [34], yet systematic reviews have highlighted the lack of exploration on other, more clinically accessible variables for dementia prediction [12, 35]. Machine learning showed added benefit only when using survival techniques, as our elastic net Cox regression outperformed the regular Cox regression. A recent comparative study on various machine learning survival models and Cox regression for dementia prediction also found similar accuracy across techniques [36], which is also in line with previous studies assessing possible performance differences between conventional regression techniques and machine learning [37, 38]. Further, a study predicting two-year incident dementia also found similar performance across traditional techniques (i.e., logistic regression) and machine learning algorithms, with a slight added benefit of machine learning models regarding positive predictive value [39]. The current study found a slight advantage over elastic net regression, which was also found in a simulation study [38]. To note, elastic net reduces the risk of overfitting by penalizing the estimates. This also increases comprehensibility of the prognostic model by decreasing the number of required variables. We were also able to build a nomogram from our elastic net Cox regression, highlighting the feasibility and explainability of using machine learning in clinical settings [40]. This study highlights the importance of censoring in risk prediction as well as the use of algorithms that can capture interactions and high-dimensional relationships within predictors, such as with machine learning [41]. Further, when removing neuroimaging markers, the performance of all models, including those using traditional statistical techniques, lowered, but remained high overall.

The most important variables for prediction in our final elastic net Cox regression included age, subjective memory complaints, and MMSE score. Subjective memory decline has been shown to be present years before mild cognitive impairment and later dementia [42], highlighting its possible use in early prediction. Further, variables such as ‘forgetting where things are’ or ‘difficulty dressing oneself’ were also present in our final model, which are items similar to those being used to create a telephonic interview for dementia prediction [43]. Functional limitations were also found in previous studies to be highly predictive of later developing dementia [44, 45]. Previous studies have explored the use of neuropsychological assessments for prognostic models of dementia [9, 46], however the current study only used the MMSE and still showed high performance. To note, the variables with most predictive power in our model were used in the three-step procedure to diagnose dementia during follow-up at the clinic, i.e., the MMSE and the ADL score, which may have induced overfitting into our model. However, our study focused on the feasibility of using machine learning methods for dementia prediction.

One recent study using population-based data from the UK Biobank also explored the use of machine learning for dementia prediction, with five and ten-year predictions [47]. However, one of the top predictors was APOE e4 genotype, making this model less clinically accessible due to the need for genotyping. APOE e4 genotype was also used in some previous prediction models, focusing on individuals already at risk (i.e., those with amnestic mild cognitive impairment) [48], and it is also included in the well-known Disease State Index (DSI) model [49]. The current study focused on the feasibility of using clinically accessible variables; therefore, we aimed to assess if performance can remain high for prediction even without genotyping.

While performing sex-stratified validation of prediction models is still quite novel and explorative, our study found differences in the elastic net Cox regression when testing our prediction model in women and men separately. As sex differences in dementia have been highlighted previously with the push for sex-based prognostic models [50, 51], future studies should further explore the possible benefit of creating sex-stratified prognostic models.

Strengths of the current study include using multiple imputation to address missing data and cross-validation to increase variability in training of the prediction models. We additionally address differences between novel machine learning classifiers, classical logistic and Cox regression, and using a survival-based machine learning method (i.e., the elastic net Cox regression). The current study also had a large sample size from a well-phenotyped, community-based population. We also report calibration, which has been highlighted as lacking in previous prognostic studies [37, 52]. Further, tuning of the machine learning classifiers was done for recalibration. We also were able to extract a clinically relevant nomogram from our elastic net Cox regression that makes our machine learning methods translatable to clinical practice. Lastly, we performed resampling and threshold adjustment which further helps address imbalanced classification.

The current study also had limitations. The models presented first need to be externally validated to assess its transportability to other populations. Further, the ascertainment of dementia was done with a three-step procedure that consisted of the ADL and MMSE, which were also used as predictors. Further, the AGES-Reykjavik cohort is predominantly White; therefore, it is crucial for the validation of this model in marginally underrepresented populations. Further, development of prognostic models in systemically minoritized groups should also be prioritized for future research. Lastly, we did not assess different time-windows for our survival models as we solely aimed to assess the comparability of techniques. Future studies should assess which models suit best for shorter- or longer-term prediction of dementia.

Our results showed that prediction models developed using supervised machine learning classifiers are feasible and add to the model’s performance, only when using survival methods. We also exemplify ways to implement machine learning in a classical point-based method using a nomogram. Additionally, model performance remained high after the removal of MRI variables. As dementia becomes a leading problem in developing countries, focusing on clinically accessible variables for the prognostication of dementia is crucial.

### Electronic supplementary material

Below is the link to the electronic supplementary material.

Supplementary Material 1

## Data Availability

Data from the AGES-Reykjavik study are available through collaboration (AGES_data_request@hjarta.is) under a data usage agreement with the IHA.
