# Supplementary material for: Dementia prediction in the general population using clinically accessible variables: a proof-of-concept study using machine learning. The AGES-Reykjavik study
Source: BMC Med Inform Decis Mak. 2023 Aug 28;23:168. doi: 10.1186/s12911-023-02244-x (PMC10463542; doi:10.1186/s12911-023-02244-x)
Supplement: Supplementary file 1 — Supplementary Material 1 [file 12911_2023_2244_MOESM1_ESM.docx]

**Supplementary Info 1.** Details on clinical variables used.

The following variables were continuous: abdominal circumference, high-density lipoprotein, low-density lipoprotein, triglycerides, fasting glucose, b-hemoglobin A1c, high-sensitive c-reactive protein, systolic and diastolic blood pressure, log-transformed morning salivary cortisol, log-transformed evening salivary cortisol, total score on the Geriatric Depression Scale-15 (GDS-15), and the carotid intima-media thickness test (CIMT).

The following variables were dichotomous: hypertension, coronary artery disease, diabetes mellitus (type 2), metabolic syndrome, stroke/blood clot in the brain, history of cancer, ever experienced a head trauma or lost consciousness, subjective cognitive decline, often forget the names of a friend, often forget where left things, difficulty finding the right words, difficulty finding the way to familiar places, ever felt intermittent claudication in legs, insomnia, Mini-International Neuropsychiatric Interview (MINI) diagnosis of current generalized anxiety disorder, social phobia, panic disorder, or agoraphobia, and the MINI diagnosis of history/current of major depressive disorder.

Categorical predictors were as follows: difficulty in managing money (no difficulty, some difficulty, much difficulty, I am unable to do it), difficulty dressing (e.g., tie, zippers, or buttons) (no difficulty, some difficulty, much difficulty, I am unable to do it), health status (excellent, very good, good, fair, poor), Activities of Daily Living (ADL) total score (dressing, bathing, transferring, eating, walking), and the total score of following anxiety questions (experienced anxiety/fright in the last 30 days, lately felt anxious/not well, or that special situations may you anxious).

**Supplemental Info 2.** Definition of all acronyms used.

| **ADL** | **Activities of Daily Living** |
| --- | --- |
| **ADNI** | **Alzheimer’s Disease Neuroimaging Initiative** |
| **AGES-Reykjavik Study** | **Age, Gene/Environment Susceptibility-Reykjavik Study** |
| **AUC** | **Area under the receiver operating characteristic curve** |
| **DSI** | **Disease State Index** |
| **MMSE** | **Mini Mental State Examination** |
| **MRI** | **Magnetic resonance imaging** |
| **TRIPOD** | **Transparent Reporting of a Multivariable Prediction Model for Individual Prognosis or Diagnosis (TRIPOD)** |

**Supplementary Code 1.** Pseudocode of algorithms used.

upsample <- trainControl(method = “repeatedcv”, number = 10, repeats = 10, classProbs = TRUE, summaryFunction = twoClassSummary, savePredictions = TRUE, sampling = “up”)

downsample <- trainControl(method = “repeatedcv”, number = 10, repeats = 10, classProbs = TRUE, summaryFunction = twoClassSummary, savePredictions = TRUE, sampling = “down”)

*Logistic regression*

Logfit <- train(outcome ~ ., training.data, metric = “ROC”, method = “glm”, trControl = upsample)

*Elastic net regression*

Elastic_net <- train(outcome ~ ., train, metric = “ROC”, method = “glmnet”, trControl = upsample, importance = TRUE)

*Random forest*

Rf <- train(outcome ~ ., train, metric = “ROC”, method = “ranger”, tuneGrid = expand.grid(mtry = c(2, 5, 10, 19), splitrule = c(“gini”, “extratrees”), min.node.size = 1), trcontrol = upsample, importance = “permutation”)

*Support vector machine*

Svm <- train(outcome ~ ., train, metric = “ROC”, method = “svmRadial”, trControl = downsample, scale = FALSE)

*Cox regression*

Coxreg <- cv.glmnet(x = variables, y = outcome, family = “cox”, nfolds = 10, type.measure = “C”)

*Elastic net Cox regression*

Enet.cox <- fit_enet(x = variables, y = outcome, rule = “lambda.1se”, seed = c(5, 7), parallel = TRUE)

**Supplementary Table 1.** Characteristics of the predictors in the study sample (n = 4793).

|  | **Train data**  **(n = 3138)** | **Test data**  **(n = 1655)** |
| --- | --- | --- |
|  | **Mean (SD) or n (%)** | **Mean (SD) or n (%)** |
| **Demographics** |  |  |
| Age (years)* ^+^ | 76 (6) | 76 (6) |
| Sex (female)* ^+^ | 1823 (58%) | 999 (60%) |
| Education (college + university) | 911 (29%) | 481 (29%) |
|  |  |  |
| **Neuroimaging variables** |  |  |
| Log-transformed white matter lesion volume (ml)* | 2.6 (0.9) | 2.6 (0.9) |
| Hippocampal volume (ml)* | 5.6 (0.7) | 5.6 (0.7) |
| Number of microbleeds* | 0.3 (1.8) | 0.3 (1.4) |
| Presence of infarcts | 961 (31%) | 530 (32%) |
| Gray matter/intracranial volume ratio* | 0.5 (0.04) | 0.5 (0.04) |
|  |  |  |
| **Clinical variables** |  |  |
| Abdominal circumference (cm) | 101 (12) | 101 (12) |
| Carotid intima-media thickness test (CIMT) | 1 (0.1) | 1 (0.1) |
| High-density lipoprotein (mmol/L) | 1.6 (0.5) | 1.6 (0.5) |
| Low-density lipoprotein (mmol/L) | 3.5 (1) | 3.5 (1) |
| Triglycerides (mmol/L) | 1.2 (0.6) | 1.2 (0.7) |
| Fasting glucose (mmol/L) | 5.8 (1.1) | 5.8 (1.3) |
| B-hemoglobin A1c (g/dl) | 0.5 (0.1) | 0.5 (0.1) |
| High-sensitive c-reactive protein (mg/L) | 3.8 (7) | 3.7 (6.3) |
| Systolic blood pressure (mmHg) | 143 (21) | 142 (20) |
| Diastolic blood pressure (mmHg) | 74 (10) | 74 (10) |
| Hypertension | 2548 (81%) | 1307 (79%) |
| Coronary artery disease | 560 (18%) | 282 (17%) |
| Diabetes mellitus | 368 (12%) | 223 (14%) |
| Metabolic syndrome | 967 (31%) | 532 (32%) |
| Stroke/blood clot in the brain | 195 (6%) | 102 (6%) |
| History of cancer | 482 (15%) | 271 (16%) |
| Experienced a head trauma or lost consciousness | 245 (8%) | 171 (10%) |
| Subjective memory decline*^+^ | 952 (30%) | 479 (29%) |
| Often forget the names of a friend | 982 (31%) | 540 (33%) |
| Often forget where items are*^+^ | 1374 (44%) | 709 (43%) |
| Difficulty finding the right words | 983 (31%) | 534 (32%) |
| Difficulty finding the way to familiar places*^+^ | 238 (8%) | 147 (9%) |
| Inability in managing money*^+^ | 74 (2%) | 58 (4%) |
| Inability in dressing oneself*^+^ | 13 (<1%) | 16 (1%) |
| Intermit claudication in legs | 148 (5%) | 79 (5%) |
| Insomnia | 963 (31%) | 527 (32%) |
| Poor health status | 181 (6%) | 95 (6%) |
| ADL score, full dependence on all items*^+^ | 28 (1%) | 24 (2%) |
| Morning salivary cortisol (nmol/L) | 19.8 (13.2) | 19.6 (13.3) |
| Evening salivary cortisol (nmol/L) | 3.9 (6.4) | 4.0 (7.4) |
| GDS-15 sum score*^+^ | 2 (2) | 2 (2) |
| All anxiety questions ‘yes’ | 23 (1%) | 17 (1%) |
| Diagnosis of current GAD, social phobia, panic disorder, or agoraphobia | 68 (2%) | 30 (2%) |
| Current/past diagnosis of major depressive disorder | 167 (5%) | 81 (5%) |
|  |  |  |
| **Medication use** |  |  |
| Benzodiazepines | 258 (8%) | 138 (8%) |
| Beta-adrenergic blockers | 1090 (35%) | 570 (34%) |
| Glucocorticoids | 108 (3%) | 63 (4%) |
| Psycholeptics | 539 (17%) | 279 (17%) |
| Anti-depressants | 427 (14%) | 235 (14%) |
|  |  |  |
| **Lifestyle variables** |  |  |
| Current smoker, % | 377 (12%) | 205 (12%) |
| Alcohol consumption (g/week) | 16 (35) | 14 (28) |
| Moderate/high physical activity | 964 (31%) | 545 (33%) |
| Mental leisure activity (days per month) | 7 (6) | 7 (6) |
| Social leisure activity (days per month) | 4 (4) | 4 (4) |
| Single marital status, % | 187 (6%) | 101 (6%) |
| Number of close friends | 3 (4) | 3 (3) |
| Not that easy to walk 2 km*^+^ | 615 (20%) | 345 (21%) |
| Not that easy to walk 500 m*^+^ | 157 (5%) | 76 (5%) |
| Number of living close relatives | 7 (5) | 7 (5) |
| Never fish consumption, % | 19 (1%) | 7 (<1%) |
|  |  |  |
| **Cognitive assessment** |  |  |
| MMSE total score*^+^ | 27 (3) | 27 (3) |
|  |  |  |
| **Outcome** |  |  |
| Incident dementia | 583 (19%) | 309 (19%) |
| Follow-up time (years) | 9 (3) | 9 (3) |

**Note:** * marks variables entered in model 2. ^+^ marks variables entered in model 3. A significant difference was found between train and test data for experiencing a head trauma or losing consciousness (χ2 = 8.4, p = 0.004), inability to dress oneself (χ2 = 9.0, p = 0.03), and ability to walk 2 kilometers (χ2 = 6.7, p = 0.03).

**Supplementary Table 2.** Tuning parameters for each machine learning classifier.

| **Classifier** | **Tuning parameter** |
| --- | --- |
| *Model 1* | |
| Elastic net | Alpha = 1, lambda = 0.02 |
| Random forest | Mtry = 5, splitrule = gini, minimum node size = 1 |
| Support vector machine | Sigma = 0.01, cost = 0.25 |
| *Model 2* | |
| Elastic net | Alpha = 0.55, lambda = 0.02 |
| Random forest | Mtry = 2, splitrule = gini, minimum node size = 1 |
| Support vector machine | Sigma = 0.05, cost = 0.25 |
| *Model 3* | |
| Elastic net | Alpha = 0.55, lambda = 0.02 |
| Random forest | Mtry = 2, splitrule = gini, minimum node size = 1 |
| Support vector machine | Sigma = 0.07, cost = 0.25 |

**Supplementary Table 3.** Elastic net and logistic regression coefficients in models 2 and 3.

|  | **Elastic net regression** | | **Logistic regression** | |
| --- | --- | --- | --- | --- |
|  | **Model 2** | **Model 3** | **Model 2** | **Model 3** |
| **Intercept** | -1.621 | -6.583 | -0.732 | -7.927 |
| **Age** | 0.070 | 0.098 | 0.082 | 0.124 |
| **Sex** | 0.034 | 0.184 | 0.288 | 0.296 |
| **Subjective cognitive decline** | 0.349 | 0.282 | 0.459 | 0.445 |
| **Difficulty in remembering where things are** | 0.221 | 0.334 | 0.300 | 0.326 |
| **Difficulty finding familiar places** | 0 | 0.050 | 0.080 | 0.278 |
| **Difficulty in managing money (some difficulty)** | 0 | 0.011 | -0.178 | -0.049 |
| **Difficulty in managing money (much difficulty)** | 0 | 0 | -0.854 | -0.741 |
| **Difficulty in managing money (unable)** | -0.120 | -0.089 | -0.722 | -0.845 |
| **Difficulty in dressing oneself (some difficulty)** | 0 | 0 | 0.205 | 0.159 |
| **Difficulty in dressing oneself (much difficulty)** | 0 | 0 | 0.199 | 0.141 |
| **Difficulty in dressing oneself (unable)** | 0.092 | 0.269 | 0.961 | 1.155 |
| **Difficulty in walking 2 km (somewhat easy)** | 0 | 0 | -0.072 | -0.093 |
| **Difficulty in walking 2 km (not very easy)** | -0.098 | 0 | -0.312 | -0.268 |
| **Difficulty in walking 500 m (somewhat easy)** | 0.132 | 0.073 | 0.317 | 0.351 |
| **Difficulty in walking 500 m (not very easy)** | 0 | 0 | 0.084 | 0.259 |
| **1 ADL item dependent** | 0 | -0.001 | -0.165 | -0.181 |
| **2 ADL items dependent** | 0 | 0 | 0.043 | 0.009 |
| **3 ADL items dependent** | 0 | 0 | -0.012 | 0.128 |
| **4 ADL items dependent** | 0 | 0.300 | -0.082 | 0.137 |
| **5 ADL items dependent** | 0.839 | 0.512 | 0.723 | 0.832 |
| **GDS-15 score** | 0 | 0 | -0.023 | -0.010 |
| **MMSE score** | -0.043 | -0.053 | -0.071 | -0.078 |
| **Log-transformed WML volume** | 0.360 | *Not included* | 0.468 | *Not included* |
| **Hippocampal volume** | -0.432 | *Not included* | -0.553 | *Not included* |
| **Microbleeds** | 0.009 | *Not included* | 0.015 | *Not included* |
| **Gray matter/ICV ratio** | -3.419 | *Not included* | -5.220 | *Not included* |

*Note:* 0s represent variables that were penalized to 0 during model fit. GDS= Geriatric Depression Scale; ICV = intracranial volume; WML = white matter lesion.

**
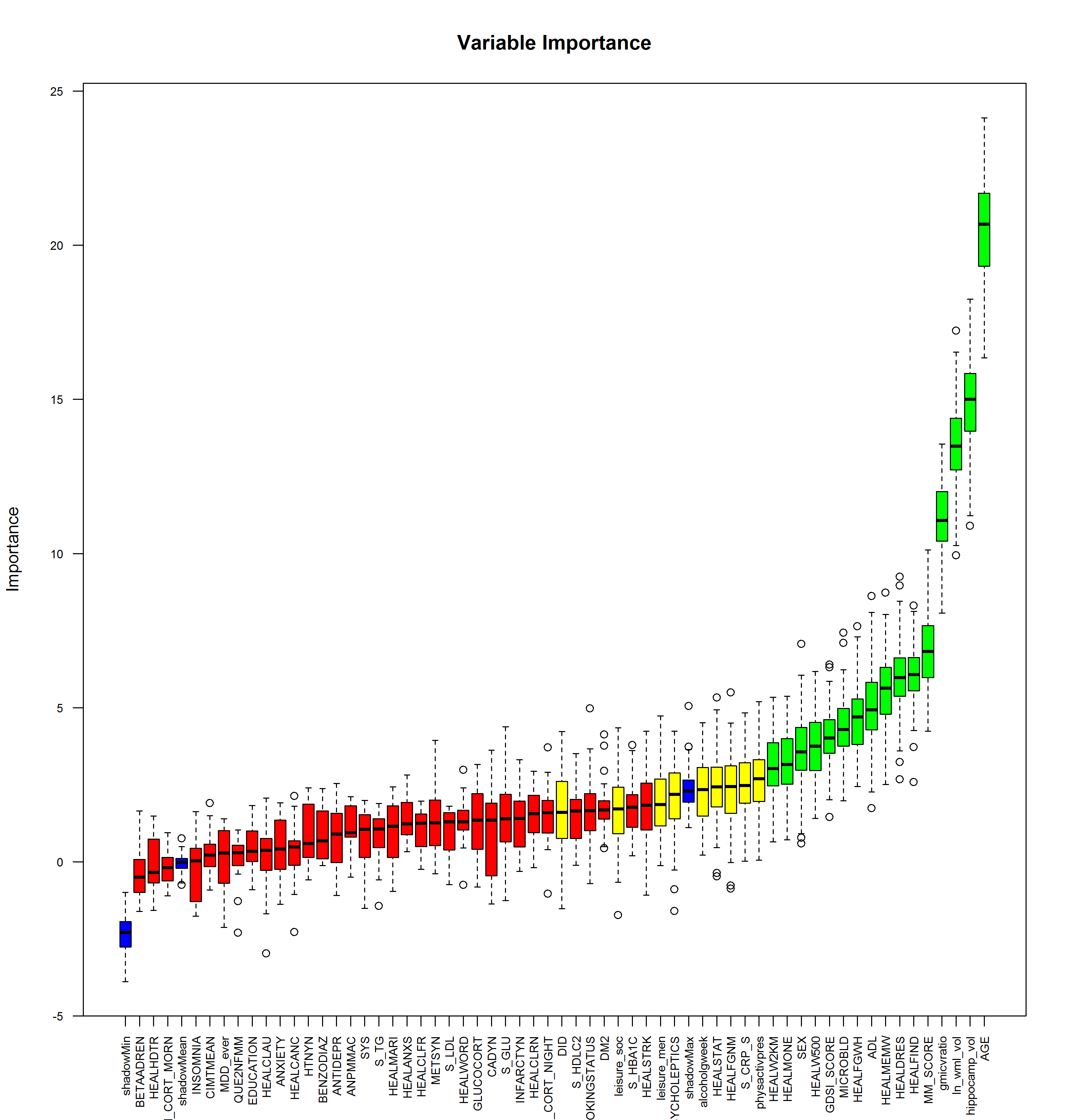
Supplementary Figure 1.** Boruta feature selection.

Green variables are selected for feature selection, yellow variables are rated as tentative, and red variables are ranked as unimportant. Ninety-nine iterations were performed.

**Supplementary Figure 2.** Variable importance per trained prognostic model in model 3, the clinically accessible model.


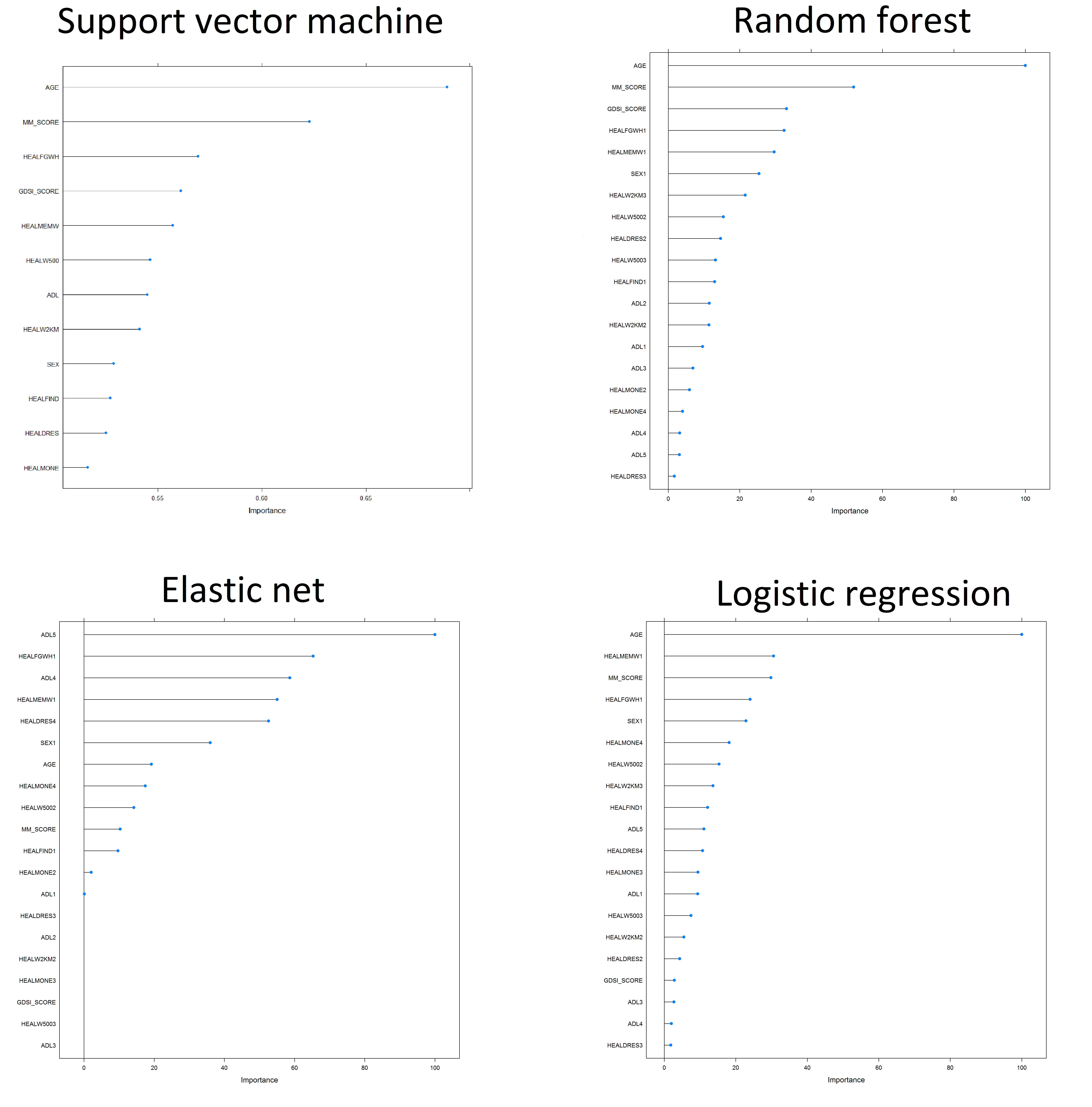


*Note:* As there is no built-in variable importance for support vector machine, the AUC is shown instead on the x-axis.
